# Anthocyanins in the Management of Metabolic Syndrome: A Pharmacological and Biopharmaceutical Review

**DOI:** 10.3389/fphar.2018.01310

**Published:** 2018-12-04

**Authors:** Rozita Naseri, Fatemeh Farzaei, Pouya Haratipour, Seyed Fazel Nabavi, Solomon Habtemariam, Mohammad Hosein Farzaei, Reza Khodarahmi, Devesh Tewari, Saeideh Momtaz

**Affiliations:** ^1^Internal Medicine Department, School of Medicine, Kermanshah University of Medical Sciences, Kermanshah, Iran; ^2^Pharmaceutical Sciences Research Center, Kermanshah University of Medical Sciences, Kermanshah, Iran; ^3^Department of Chemistry, Sharif University of Technology, Tehran, Iran; ^4^Phyto Pharmacology Interest Group, Universal Scientific Education and Research Network, Los Angeles, CA, United States; ^5^Applied Biotechnology Research Center, Baqiyatallah University of Medical Sciences, Tehran, Iran; ^6^Pharmacognosy Research Laboratories, Medway School of Science, University of Greenwich, Kent, United Kingdom; ^7^Medical Biology Research Center, Kermanshah University of Medical Sciences, Kermanshah, Iran; ^8^Department of Pharmaceutical Sciences, Faculty of Technology, Kumaun University, Nainital, India; ^9^Medicinal Plants Research Center, Institute of Medicinal Plants, ACECR, Karaj, Iran; ^10^Toxicology and Diseases Group, The Institute of Pharmaceutical Sciences, Tehran University of Medical Sciences, Tehran, Iran

**Keywords:** anthocyanins, natural pigments, phytochemicals, metabolic syndrome, diabetes mellitus, insulin resistance

## Abstract

The term “metabolic syndrome” (MetS) refers to a combination of diabetes, high blood pressure, and obesity. The origin of MetS includes a combination of multiple factors, such as sedentary lifestyle, unhealthy diet choice, and genetic factors. MetS is highly prevalent and adversely affects the general population by elevating risk of cardiovascular complications, organ failure, and much other pathology associated with late-stage diabetes. Anthocyanins (ANTs) are health-promoting bioactive compounds belonging to the flavonoids subclass of polyphenols. Numerous studies have reported the potential therapeutic benefits on MetS syndrome and diabetes from fruits rich in ANTs. This review summarizes the role of several dietary ANTs on preventing and managing MetS as well as the pharmacological mechanisms and biopharmaceutical features of their action. We also discuss potential nanoformulation and encapsulation approaches that may enhance the bioefficacy of ANTs in MetS. Experiments have demonstrated that ANTs may attenuate the symptoms of MetS via improving insulin resistance, impaired glucose tolerance, dyslipidaemia, cholesterol levels, hypertension, blood glucose, protecting β cells, and preventing free radical production. In brief, the intake of ANT-rich supplements should be considered due to their plausible ability for prevention and management of MetS. Additionally, randomized double-blind clinical trials are obligatory for evaluating the bioefficacy and pharmacological mechanisms of ANTs and their pharmaceutical formulations in patients with MetS.

## Introduction

Metabolic syndrome (MetS), also known by other names such as “insulin resistance syndrome” or “syndrome X,” was first defined by Kylin in the 1920s as a combination of hyperglycemia, hypertension, and gout. To date, its diagnostic criteria include atherogenic dyslipidemia [low HDL cholesterol and high triglycerides (TGs)], hyperglycemia, insulin resistance (landmark sign of the disease), glucose intolerance, hypertension, and central obesity (Spiegelman and Flier, 2001; Ford et al., 2002; Grundy et al., 2004; Alberti et al., 2005; Russell and Proctor, 2006; Romeo et al., 2012). Hence, MetS is common medical terminology for a combination of diabetes, high blood pressure, and obesity. Furthermore, central obesity or hypertriglyceridaemic waist phenotype contributes to the development of hyperinsulinemia, lipid abnormalities, hyperglycemia, and the activation of inflammatory and prothrombotic mediators with an amplified risk of the prevalence of type 2-diabetes mellitus (DM), cardiovascular diseases (CVD), and many cancers (Berlin et al., 2000; Festa et al., 2001; Lakka et al., 2002; Carr et al., 2004; Gluckman and Hanson, 2004; Hansel et al., 2004; Guilder et al., 2006; Vlachopoulos et al., 2010). DM is a chronic disease diagnosed by hyperglycemia, owing to insufficient insulin production or inadequate cellular sensitivity to insulin, and progressive decline in B-cell function (Kudva and Butler, 1997; American Diabetes Association, 2009). DM is a rising global problem and expected to affect around 380 million by 2025 (Kaul et al., 2013). Several studies suggested that plant derivatives such as polyphenols possess numerous biological activities with anti-inflammatory, antioxidative, and insulin-sensitizing effects (Hämäläinen et al., 2007; Shamim, 2009; Sodagari et al., 2015). Natural supplements and various herbal products, especially ANT-rich food, are claimed to be beneficial in controlling MetS. Thus, in the present review, we reviewed ANT-rich food as potential alternative therapeutic as well as their possible mechanisms of action for managing MetS.

## Epidemiology of Metabolic Syndrome (MetS)

The worldwide prevalence of MetS varies between 10 and 84% for urban populations based on the region, composition (age, sex, race) of the population, and the definition of MetS. The International Diabetes Federation (IDF) estimated that approximately one-quarter of the global adult population has MetS, of which 28% were men and 34% were women belonging to the atherosclerosis risk in communities (ARIC) study population (Desroches and Lamarche, 2007; Kolovou et al., 2007). In a survey conducted on 8,814 people in the USA, the prevalence of MetS was more than 40% in population between 60 and 69 years (Ford et al., 2002; Day, 2007). According to Amirkalali et al. the prevalence of MetS in Iranian individuals was 36.9%, depending on the adult treatment panel III (ATP III) criteria, 34.6% according to the IDF, and 41.5% based on the Joint Interim Societies (JIS) criteria (Amirkalali et al., 2015). The high prevalence of MetS is responsible for substantial public health consequences owing to augmented risk of type 2 DM and CVD (Carr et al., 2004). Nowadays, diabetes is becoming a global pandemic with increasing prevalence in India and Asia, whom will be the 7th leading reason of death by the year 2030 according to the World Health Organization (WHO) estimates (World Health Organization, 2012; Maiese, 2015; Munasinghe and Katare, 2016).

## Current Therapeutic Protocols For MetS

The primary cause of MetS is diet, obesity, physical inactivity, age, and genetic profiles, such as a defect in a single gene, in lamin A/C, O-acyltransferase, 1-acylglycerol-3-phosphate, seipin, the adrenergic receptor, and adiponectin (Steppan et al., 2001; Lakka and Laaksonen, 2007; Schröder, 2007; Abete et al., 2011; Kastorini et al., 2011; Amiot et al., 2016; Martinez-Abundis et al., 2016; Merone and McDermott, 2017). Evidence indicates that combination of lifestyle modifications with effective weight loss and drug therapy may serve as treatment for MetS (Marvasti and Adeli, 2010). First-line recommendations include lifestyle modification as well as introduction of the Mediterranean diet, which includes more fruit and vegetable consumption along with higher monounsaturated fat intake (Esposito et al., 2004). Such an approach may suppress the postprandial glycaemia, serum TG levels, and raise HDL-cholesterol; thus, delaying the transition from impaired glucose tolerance to incidence of type 2 DM, and reducing risk of developing MetS (Tsuda, 2012).

Since insulin resistance plays significant role in regulating diabetes, pharmacological interventions, such as thiazolidinediones and metformin, seem to have supplementary effects in ameliorating diabetes and/or MetS evolution by stimulating muscle glucose uptake and suppressing hepatic glucose production along with AMP-activated protein kinase (AMPK) activation. AMPK is a major cytological regulator of glucose and lipid metabolism, thereby is considered as a potential target for therapeutic management of type 2 DM (Zhou et al., 2001; Hawley et al., 2002; Grewal et al., 2016; Maskimov et al., 2016).

Lipid-lowering agents and low-density lipoprotein (LDL) lowering standard drugs, such as statins and ezetimibe, modify atherogenic dyslipidemia, and CVD in patients with MetS. Other drugs that reduce MetS progression include thiazolidinediones, glucagon-like peptide-1 (GLP-1) agonists, and inhibitors of dipeptidyl peptidase-4(DPP-4). Once statin therapy and lifestyle modifications are not successful, niacin may be helpful to reduce TG (Marvasti and Adeli, 2010).

To manage diabetes, there are several ongoing drug therapy approaches including sulphonylureas, metformin, and α-glucosidase inhibitors, which suppress and interfere with gut glucose production and absorption, but may become refractory to the treatment over time. It is now clear that the aggressive control of hyperglycaemia by synthetic drugs in patients with MetS may be involved in the progression of various chronic complications, such as retinopathy and nephropathy. Since the utilization of oral antihyperglycemic drugs have limited efficacy and numerous side effects, complementary and alternative medicines such as acupuncture, herbal medicines, Ayurveda, traditional medicine, and other medicinal approaches may be helpful in the management of MetS.

## Molecular Pathophysiology of MetS

**Figure 1:**
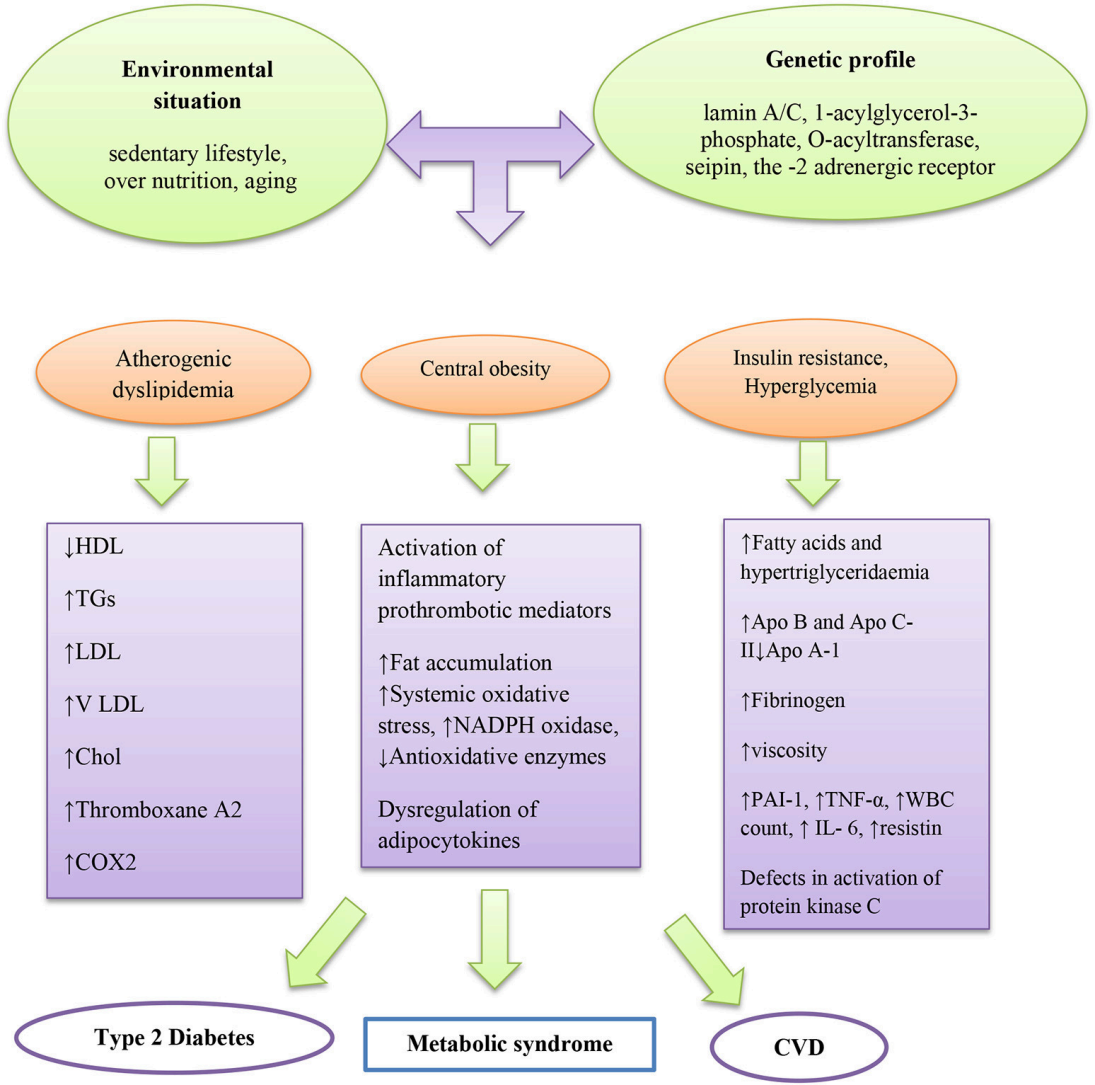
Main pathogenic mechanism of metabolic syndrome.

Diverse pathophysiologic factors that may drive the progression of MetS, for instance, insulin resistance with circulating fatty acids accumulation and adiposity are the main factors (Montague and O'rahilly, 2000; Taniguchi et al., 2006; Barazzoni et al., 2018) (Figure 1). Insulin resistance is a physical condition, which is demarcated as a state that needs additional insulin to produce biological effects with decreasing glucose uptake in muscle and adipose tissue. Insulin affects antilipolysis and stimulates lipoprotein lipase via inhibition of lipolysis in adipose tissue. Therefore, when insulin resistance occurs, increasing amounts of fatty acids are produced by high amounts of stored triacylglycerol molecules, inciting additional lipolysis in adipose tissue. In the liver, insulin resistance leads to flaws in insulin receptor substrate-1 and substrate-2 tyrosine phosphorylation, leading to the activation of protein kinase C. Excessive fatty acids may also impair activation of protein kinase C as well as acyl-coenzyme A (CoA) generation in muscles.

Obesity is linked with accumulation of higher macrophages in adipose tissues and augmented pro-inflammatory cytokines (Di Gregorio et al., 2005). Fat accumulation in adipose tissue, liver, skeletal muscle, heart, and pancreas may increase systemic oxidative stress independent of hyperglycemia (Unger, 2003) as well as adipocytokines or adipokines such as plasminogen activator inhibitor−1 (PAI-1), tumor necrosis factor (TNF)-α, resistin, and leptin (Friedman and Halaas, 1998; Leyva et al., 1998; Matsuzawa et al., 1999; Niemann et al., 2017; Reho and Rahmouni, 2017; Louwen et al., 2018). In a research conducted by Guzmán-Gerónimo et al. a high-fat diet caused increased arterial blood pressure, high levels of TG in plasma, and reduction of HDL-C due to elevation of fatty acid reesterification (Guzmán-Gerónimo et al., 2017). Likewise, the reduction of HDL-C has been reported in humans with MetS (Guzmán-Gerónimo et al., 2017).

## Role of Oxidative Stress in Pathogenesis of MetS

Oxidative stress, a shift of the redox balance, is a deleterious condition that occurs when cellular components including proteins, lipids, and DNA are damaged. Selective radical overgeneration in adipose tissue may possess a prominent role in insulin resistance development, diabetes, and CVD through impairment of muscle glucose uptake and secretion of insulin from β cells (Maddux et al., 2001). Numerous studies revealed that an increased level of reactive oxygen species (ROS) in peripheral blood from accumulated fat is involved in initiation of insulin resistance in different adipose tissue, skeletal muscle, and other diabetic complications. Insulin resistance leads to disruption of many prime oxidative reactions, resulting in undue ROS generation at cellular and mitochondrial levels. Studies have shown that in type 2 diabetic patients, lipid peroxidation increased, while the levels of plasma glutathione (GSH) and GSH-metabolizing enzymes are reduced (Sundaram et al., 1996). Folmer et al. reported that hyperglycemia induces free radical and oxidative stress production in mice (Folmer et al., 2002). An application of about 10–20 mM glucose into the posterior root ganglion neurons resulted in production of O${}_{2^{-}}$, H_2_O_2_, lipid oxidation and neuronal death (Schmeichel et al., 2003).

Adiponectin, an anti-inflammatory cytokine produced by adipocytes, improves insulin sensitivity and inhibits many inflammatory processes. In cultured adipocytes, oxidative stress was increased when the level of fatty acid was enhanced, this was attributed to the activation of NADPH oxidase and generation of adipocytokines (fat-derived hormones) at a deregulated manner (Hotamisligil et al., 1993; Shimomura et al., 1996; Lara-Castro et al., 2006). NADPH oxidase is a key source of ROS production in adipocytes, which increases in obesity. Treatment with NADPH oxidase inhibitor may decrease ROS production in adipose tissue, attenuate adipocytokines dysregulation, and ameliorate hyperlipidemia and diabetes in obese mice, also may reduce pathogenesis of several vascular diseases like hypertension and atherosclerosis (Iwaki et al., 2003; Farzaei et al., 2017, 2018; Furukawa et al., 2017).

## Anthocyanins as Antioxidant

**Figure 2:**
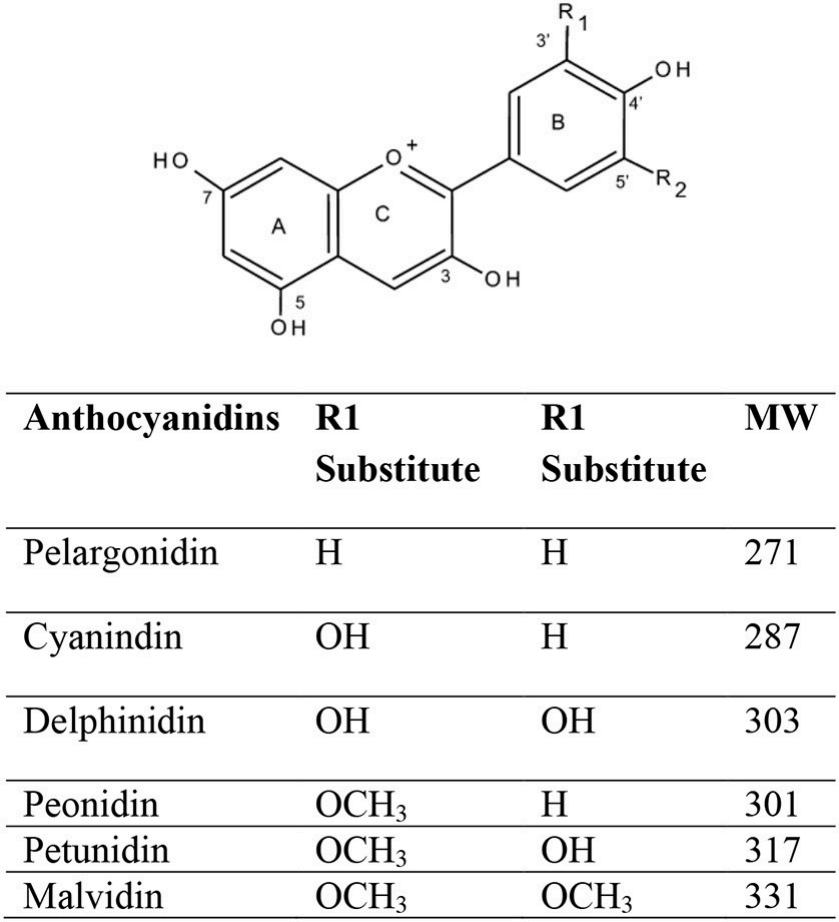
Structures of common anthocyanidins in fruits and vegetables.

The term “Anthocyanin” is derived from two Greek words, i.e., antos for flower and kyanos means blue. ANTs are one of the most important health-promoting natural plant pigments, which belong to the flavonoids group and polyphenol class of phytochemicals (Dreiseitel et al., 2008; Pojer et al., 2013). Variations in ANTs are a result of the number and degree of methylation, hydroxyl group position, and the number of rings (aliphatic/aromatic) that are attached to the sugar moieties, also are dependent on the location and type of sugar attachment to the molecule on the basic anthocyanidin skeleton (Deng et al., 2013). Flavylium cation (2-phenylbenzopyrilium) is the fundamental structure that links with either one or more sugar moiety and hydroxyl (-OH) and/or methoxyl (-OCH_3_) groups. Cyanidin-3-glucoside, cyanidin-3-(xylose-glucose-galactoside), cyanidin-3-(xylose-feruloyl-glucose-galactoside), cyanidin-3-(xylose-sinapoyl-glucose-galactoside), cyanidin-3- (xylose-galactoside), and cyanidin-3-(xylose-coumuroyl-glucose-galactoside are some of the most important ANTs (Table 1, Figure 2).

**Table 1:** Major anthocyanidins in plants (Pojer et al., 2013; Fang, 2015).

**Selected plant source**	**Anthocyanidins**
Apple, elderberry, blackberry, pear, peach, fig, cherry, red onion, red cabbage, rhubarb, gooseberry	Cyanidin
Banana, red radish, strawberry, potato	Pelargonidin
Pomegranate, black currant, gooseberry, purple carrot, blood orange, egg plant, green bean	Cyanidin and delphinidin
Pomegranate, passion fruit, eggplant, green bean	Delphinidin
Plum, sweet cherry, purple sweet potato	Cyanidin and peonidin
Mango	Peonidin
Bilberry, red grape	Petunidin and malvidin

ANTs are water-soluble bioactive compounds widely found in various vegetables and fruits, including berries like cranberries, strawberries, blueberries, blackberries, elderberries, grapes, currants, plums, cherries, red cabbage, red onions, and sweet potatoes. They are usually distributed in fruits and flowers; however, stems, leaves, and roots of some plants also contain different types of ANTs (Wu and Prior, 2005).

ANTs are natural antioxidants with high reactivity toward ROS, mainly due to their ability to transfer electrons or to donate the hydrogen atoms from various hydroxyl groups to free radicals, to their basic structural compounds and ring orientation, and to the unpaired electron supporting ability of ANTs (Wang et al., 1999; Anderson and Jordheim, 2008; Markakis, 2012). ANTs also have prominent therapeutic effects like anti-inflammatory, anti-viral, anti-carcinogenic, anti-mutagenic, anti-allergic, anti-microbial, improvement of arterial stiffness and antioxidants effects, and are strong lipid peroxidation inhibitors (Kim et al., 2006; Tsang et al., 2018). The antioxidant capacity of ANTs has been proven by several methods, such as oxygen radical absorbance capacity (ORAC), DPPH (2,2-diphenyl-1-picrylhydrazyl) assay, ABTS [2,2′-azino-bis(3-ethylbenzothiazoline-6-sulphonic acid)] assay, and etc. (Burns et al., 2000; Wang and Lin, 2000; Prior et al., 2001; Zheng and Wang, 2003; Steed and Truong, 2008; Sodagari et al., 2015; Yue et al., 2018).

## Biopharmaceutical Features of Anthocyanins in Metabolic Syndrome

Although ANTs have low absorption and high metabolism, the regular intake of ANTs may have critical and/or beneficial effects on human health. ANTs are absorbed intact as glycosides and their absorption rates are influenced by their chemical structures. ANTs are poorly absorbed after oral administration (about 10–50 nM) in the stomach and small intestine, and the maximal plasma concentration time is about 1.5 h. Individual ANTs absorption efficiency is between 0.12 and 0.25% for non-acylated ANTs, and 0.0079–0.019% for acylated ANTs. The acylated types showed lower affinity for the transporter bilitranslocase (Kay, 2006; Czank et al., 2013; Fernandes et al., 2014; Kay et al., 2017).

Elimination from plasma differs based upon ANTs structures. Non-acylated compounds are eliminated slower compared to the acylated forms. Additionally, variations in ANTs interactions with the transporters (at tissue level) may be responsible for the differences in the plasma kinetics. Several studies have revealed that ANTs are absorbed and excreted intact. Pharmacokinetics data analysis in animal and human subjects suggested that the intestine is the major site of ANTs absorption (Ferrars et al., 2014; Kamiloglu et al., 2015). ANTs are metabolized to glucurono-, sulfo-, or methyl-derivatives in the proximal gastrointestinal (GI) tract like other flavonoids. ANTs clearance from the circulation is suitably rapid (Bub et al., 2001).

The majority of ingested ANTs appears to reach the lower intestine, and are subjected to the microbial catabolism. In intact functioning colon volunteers, a portion of ANTs pass into the large intestine, where it is deglycosylated and the subsequent aglycones are broken down via C-ring fission with fragments of A- and B-ring (González-Barrio et al., 2011).

The investigation of the biopharmaceutical profile and comprehensive researches on different aspects of ANTs bioavailability such as absorption, distribution, metabolism, and excretion (ADME) are gaining tremendous interests recently. Diverse research groups are focusing on various ANTs to enhance their bioavailability against different diseases including cancer (Mueller et al., 2018; Thibado et al., 2018). Animal studies showed that ANTs are mainly absorbed in the intact glycosidic form and reach systemic circulation within 0.25–2 h. ANTs from *Vaccinium myrtillus* (400 mg/kg) reached peak plasma concentrations level (2–3 μg/mL) after 15 min and then, declined rapidly within 2 h upon a single oral administration in rats (Huang et al., 2014). After oral administration of cyanidin-3-glucoside (C3G) at 400 mg/kg, the intact form was rapidly observed in the plasma [Cmax: 0.31 μmol/L (0.14 μg/mL)] at 30 min (Feshani et al., 2011; Kalt et al., 2017; Tymchuk et al., 2017).

Most ANTs, especially from berries, are believed to have low bioavailability (Kay et al., 2017). The peak plasma concentration of ANTs from berries ranges between 1 and 120 nmol/L with < 1% urinary recovery confirmed by different studies (Kay, 2006) and around 0.005% level at excretion (Stalmach et al., 2012). Metabolism of ANTs occurs by the formation of sulfo-, glucurono-, or methyl-derivatives in the proximal GI tract. Unmetabolized compounds have also been observed in small quantities in the urine and systemic circulation, even though the exact mechanism for absorption is still highly theoretical (Kamiloglu et al., 2015). Several studies reported that the possible transport mechanism is through intestinal glucose transporters, stomach transporters, and tight junction permeability (Passamonti et al., 2003).

In a study conducted on 9 volunteers, 300 g raspberry with 292 μmol ANTs constituted of cyanidin-based components were ingested, and the results showed that only cyanidin-*O*-glucuronide and cyanidin-3-*O*-glucoside were traced with sub-nmol/L peak concentrations (Cmax) in the plasma, with a Tmax (Time of Peak Concentration) after 4 and 1 h, respectively. After 0–24 h, only 20 nmol of cyanidin-3-*O*-glucoside was detected in the urine and no other parent ANTs were observed (González-Barrio et al., 2010; Ludwig et al., 2015).

The findings of Felgines and colleagues demonstrated that the excretion of blackberry ANTs in urine occurs as intact and methylated forms with no conjugated or aglycones compounds. Moreover, low amounts of aglycones and ANTs were detected in cecal contents, which suggested microflora adaptation to ANTs degradation. Additionally, ANTs and their metabolites were detected in bile rapidly after oral intake, demonstrating the rapid absorption and metabolism (Felgines et al., 2002). Methylated ANTs were also recorded in rat plasma (Ichiyanagi et al., 2004). In a recent study, consumption of ANT-enriched beverages in milk and water was examined in order to investigate the role of milk on the oral bioavailability of ANTs. The authors recorded the significant effect of milk compared to water on decreasing the C_max_, the area under curve (AUC) of two individual pelargonidin ANTs (pelargonidin-3-glucuronide and pelargonidin-3-*O*-rutinoside), and C_max_ of pelargonidin-3-*O*-glucoside. The oral bioavailability of these ANTs decreased in the subjects that consumed beverages in milk by about 50% (Xiao et al., 2017). Oral administration of 100 mg delphinidin-3-glucoside/kg attained the Cmax in 15 min while the methylated form of delphinidin-3-glucoside showed C_max_ after 1 h, and the presence of ANTs glucuronides in rat plasma suggested that metabolites are produced in the liver, rather than by intestinal flora (Ichiyanagi et al., 2005).

## Role of Nanoformulation and Encapsulation Methods in Anthocyanins Bio-Efficacy For Management of Metabolic Syndrome

Various ANTs are used in the food industries as an active ingredient, but their degradation is possible after exposure to different factors such as oxygen or light, thus, stability is of prime importance when ANTs are used as a colorant in the food industry. To overcome this problem, microencapsulation is a potential technique (Favaro-Trindade et al., 2010; Nayak and Rastogi, 2010). ANTs present in pomegranate juice were relatively degraded faster in the fresh pomegranate juice than in microencapsulated powder, representing the importance of encapsulation techniques in the preservation of the bioactive compounds (Robert et al., 2010).

A number of polyphenols do not absorbed in GI track of human (Cerdá et al., 2004; Seeram et al., 2006). Therefore, nanoencapsulation may help to conquer the susceptibility of these compounds toward GI hydrolysis, low systemic bioavailability, poor absorption, and short half-life (Shirode et al., 2015). One such example is nano-pelargonidin, which enhanced protection at ~10-fold decreased dose, and is postulated to be used in the formulation of protective drugs for mitochondrial dysfunction management that is often tested in alloxan-induced hyperglycemic L6 cells (Samadder et al., 2017). Nanoformulations can improve drug delivery and bioavailability to the target cells due to their physicochemical properties, making them viable in successfully curing deadly diseases. The same research group evaluated the preventive effects of nanoencapsulated pelargonidin against alloxan-induced DNA damaged cells (L6) by *in vitro* methods, and reported around ~10-fold enhancement in efficacy of nanoencapsulated pelargonidin than pelargonidin (Samadder et al., 2016). Extracts of Chinese herbal medicine named “Shanzhuyu” containing ANTs were also used for the preparation of metal nanoparticles, and unveiled promising anticancer activity against human liver cancer (HepG2) and human prostate cancer (PC-3) cell lines (He et al., 2006, 2017). Apart from the nanoformulations and encapsulation, nano packing is also considered an emerging technique for the preservation of quality in ANT-rich fruits like strawberries (Yang et al., 2010). Although, biocompatible and safe nanoformulation is potentially important and an emerging field to enhance the bioavailability of the ANTs, only limited studies have been conducted in this regard. Therefore, nanoencapsulation and preparation of different nanoformulations targeting metabolic syndrome is in need.

## Pharmacological Mechanisms of Anthocyanins in Metabolic Syndrome

### Anthocyanins Enriched Extracts

All of ANT-enriched extracts may contain a significant amount of other non-ANT phenolics (flavonoids and/or phenolic acids) and other non-polyphenolic compounds, which may possess favorable impact/effect on the pathogenesis of the MetS. However, below is the pharmacological mechanisms of plant extracts, in which ANTs are considered as the main bioactive and major constituents.

### Berry Anthocyanins

#### Blueberry Anthocyanins

Blueberries (*Vaccinium myrtillus*) from Ericaceae family are particularly high in anthocyanidins, chlorogenic acid, flavonids, *a*-linolenic acid, pterostilbene and resveratrol. Myrtocyan is a highly purified extract of *Vaccinium myrtillus*, which contains 36% anthocyanosides including 3-arabinoside, delphinidin 3-galactoside, delphinidin, petunidin 3-arabinoside, petunidin 3-galactoside, cyanidin 3-galactoside, cyanidin 3-glucoside, cyanidin 3-arabinoside, malvidin 3-galactoside, malvidin 3-glucoside, peonidin 3-glucoside, peonidin, 3-galactoside, peonidin 3-arabinoside, and peonidin 3-glucoside (Routray and Orsat, 2011).

Malvidin-3-glucoside possesses anti-inflammatory activity in endothelial cells through inhibition of production of monocyte chemotactic protein-1 (MCP-1), intercellular adhesion molecule-1 (ICAM-1), and vascular cell adhesion molecule-1 (VCAM-1) both in protein and mRNA levels (Huang et al., 2014).

Numerous investigations indicated that blueberries have several beneficial therapeutic properties, such as attenuating age-induced oxidative stress and inflammatory responses (Lau et al., 2007), protecting the kidney (Elks et al., 2015), preventing diabetes (Martineau et al., 2006), protecting against cardiovascular disorders, preventing hyperlipidemia and hypertension (Kalea et al., 2009), and reducing obesity *in vitro* and *in vivo* (Seeram et al., 2002; Kumar et al., 2012).

Blueberries exhibited anti-inflammatory activity *in vitro* via attenuation of the balances of pro-inflammatory cytokines in lipopolysaccharide (LPS)-induced RAW264.7 macrophages (Table 2). In addition, wild blueberry-enriched diet has protective effects on the pro-inflammatory status related to the MetS in the obese Zucker rat by suppressing liver expression of NF-κB and increasing adiponectin expression (Table 3) (Seeram et al., 2002; Vendrame et al., 2013). Moreover, cellular and animal models of oxidative stress have also been utilized to prove the antioxidant potential of blueberries (Sellappan et al., 2002).

**Table 2:** *In vitro* studies evaluating the protective and therapeutic effects of anthocyanins in metabolic diseases.

**Anthocyanins**	**Cell culture model**	**Results**	**References**
Cyanidin-3-*O*-β-glucoside chloride or Cyaniding chloride	HK-2 cells	↑Cholesterol efflux & ABCA1 expression ↑PPARα & LXRα expressions ↓ICAM1,MCP1,TGFβ1 & NFκB	Du et al., 2015
Malvidin-3-Glucoside and Malvidin-3-Galactoside	HUVEC cells	↓ICAM1,MCP1 &VCAM1 ↓IκBα degradation Blocking block the nuclear translocation of p65	Huang et al., 2014
Delphinidin 3-sambubioside-5-glucoside (D3S5G)	H4IIE hepatoma cells	↓IκBα degradation ↓Gluconeogenic enzyme, glucose-6-phosphatase	Rojo et al., 2012
Pelargonidin	L6 skeletal muscle cell	↑Intracellular glucose uptake ↓GLUT4, IRS1, IRS2, PI3,GK & PK	Samadder et al., 2017
Pelargonidin	L6 cells	↓Oxidative damage Activation of DNA repaired cascades	Samadder et al., 2016
Cyanidin-3-glucoside	MIN6N pancreatic β-cells	↓Overproduction of reactive oxygen species ↓Apoptosis of cell (under high glucose condition) ↑Insulin secretion	Lee et al., 2015
Cyanidin-3-*O*-b-glucoside	THP-1 cells	↓TNFa & IL-6 expression and secretion Blockage of phosphorylation of IκBα and NF-κB nuclear translocation	Zhang et al., 2010
Blueberries and Concord grapes (containing malvidin, petunidin, or peonidin)	Mouse embryonic fibroblast cell line 3T3-L1	↑Basal oxygen consumption rate ↓Lipid accumulation ↑Mitochondrial respiration	Skates et al., 2017
Bilberry extracts	3T3-L1 cells	Inhibition of 3T3-L1 cells differentiation ↓ PPARγ ↓Sterol regulatory element-binding protein 1c (Srebp1c) ↓Phosphorylation of tyrosine residues of IRS1	Suzuki et al., 2011

*HUVECs, Human umbilical vein endothelial cells; PPARα, Peroxisome proliferator-activated receptor alpha; LXRα, Liver X receptor alpha; ICAM1, Intercellular adhesion molecule-1; MCP1, Monocyte chemoattractant protein-1; TGFβ1, Transforming growth factor-β1; MCP-1, Monocyte chemotactic protein-1; ICAM-1, Intercellular adhesion molecule-1; VCAM-1, Vascular cell adhesion molecule-1; GLUT4, Glucose transporter 4; GK, Glucokinase; PK, Pyruvate kinase*.

**Table 3:** *In vivo* studies on animal models evaluating the protective and therapeutic effects of anthocyanins and anthocyanin-rich extracts in metabolic diseases.

**Anthocyanin**	**Animal model**	**Results**	**References**
Black chokeberry extract	STZ-induced diabetes in rats and mice	Antidiabetic & hypoglycemic effect by ↑insulin secretion, maintaining the round shape of the pancreas, protecting pancreatic β cells, ↓sucrase & maltase activity, ↓LDL-cholesterol and TG	Jurgonski et al., 2008; Kim et al., 2013; Jeon et al., 2018
Black chokeberry anthocyanins	STZ -induced oxidative stress in male wistar rats	↓Body weight gain, ↓lipase, ↓pancreatic amylase & ↓carbohydrates absorption in the digestive system	Qin and Anderson, 2012
Blueberries anthocyanin	Obesogenic diet mice	↓Abdominal fat mass, ↓total fat mass, ↓body weight gain, ↓TGs, ↓liver weight, ↑PPAR	Seymour et al., 2008; Prior et al., 2010
Blueberries anthocyanin	High-fat diet induced weight gain in Male C57BL/6 mice	↓Insulin resistance, hyperglycemia ↓adipocyte death, ↓body fat accumulation & ↓body weight gain	Defuria et al., 2009; Basu et al., 2010
Blueberries anthocyanin	Cyclophosphamide-induced cardiac injury in rats	Attenuates cardiac injury by ↑heart rate & activities of heart enzymes, ↓IL-1β & TNF-α expression, ↑IL-10	Liu et al., 2015
C3G	High-fat diet induced body fat accumulation C57BL/6J mice	↓Hyperglycemia, ↓blood glucose level & modulates insulin ↓Body fat accumulation via ↓lipid synthesis in the liver and white adipose tissue	Tsuda et al., 2003
*Hibiscus sabdariffa*	High-fat diet induced obesity and liver damage in hamsters	↓Body weight, fat content & liver fat bodies ↓LDL-C & ↓TGs ↓ALT & ↓AST	Huang et al., 2015
Maqui Berry	Diet-induced obese hyperglycaemic C57BL/6J mice	↓Fasting blood glucose levels & glucose tolerance	Schreckinger et al., 2010; Rojo et al., 2012
Mulberry	High-fat diet in db/db mice	↓Fasting blood glucose, serum insulin, ↓leptin, ↓TGs & cholesterol levels ↓and LDL values ↑adiponectin levels	Yan et al., 2016
Pelargonidin	STZ-induced oxidative stress in diabetic neuropathic rat	↑SOD, malondialdehyde, fructosamine & catalase, ↑thiobarbituric acid reactive substances formation, ↓elevated blood glucose levels	Roy et al., 2008; Mirshekar et al., 2010
Pomegranate seed oil	High-cholesterol diet fed male rats	↓Weight raises & ↓body fat mass	Vroegrijk et al., 2011
Purple sweet potato anthocyanin	STZ-induced insulin deficiency in yellow db/db mice	Induced hypoglycemic activity via ↓oral glucose insulin sensitivity	Ludvik et al., 2004
Purple sweet potato anthocyanin and diacylated ANTs	STZ-induced insulin deficiency in obese Zucker fatty rats	Improved glucose tolerance & diabetes signs via ↓hyperinsulinemia & ↓hyperlipidemia as well as ↓TGs & ↓FFA, ↓maltase and ↓maximal blood glucose level & serum insulin secretion	Kusano et al., 2001
Red onion extract	Diet-induced obese hyperglycaemic C57BL/6J mice	↑Insulin sensitivity via upregulation of energy expenditure & biogenesis of mitochondrial skeletal muscle	Morrison et al., 2015
Red onions	High-fat diet in C57BL/6J mice	Attenuated hyperglycemia & ↑insulin sensitivity via ↑energy expenditure and biogenesis of mitochondrial skeletal muscle, ↑glucose tolerance, protecting DNA from oxidative stress o	Eldin et al., 2010; Jung et al., 2011
Sweet potato	STZ-induced insulin deficiency	↓Hyperlipidemia, ↓TGs & FFA ↓maximal blood glucose level & serum insulin, ↓oral glucose insulin sensitivity ↓maltase inhibitory activity	Kusano and Abe, 2000; Matsui et al., 2002

*STZ, Streptozotocin; LDL, Low-density lipoprotein; IL, Interleukin; FFAs, Freefattyacids; PPAR, Peroxisome proliferator-activated receptors; TGs, Triglycerides; TNF, Tumor necrosis factor; SOD, Superoxide dismutase; ALT, Alanine transaminase; AST, Aspartate transaminase*.

An *in vivo* study showed that blueberry reduced TGs, body weight gain, liver weight, abdominal fat mass, total fat mass, and improved adipose and skeletal muscle peroxisome proliferator-activated receptors (PPARs) activities that are involved in glucose uptake/oxidation and fat oxidation in obese rats (Table 3) (Seymour et al., 2008). Furthermore, purified blueberry and blueberry juice ANTs attenuated obesity development, elevated serum leptin, and diabetes in mice fed with obesogenic diet (Wallace et al., 2001; Prior et al., 2010).

Blueberries improve hyperglycemia, regulate skeletal muscle glucose uptake, and decrease liver glucose production *in vivo* (Defuria et al., 2009). DeFuria et al. also reported that blueberries consumption attenuated insulin resistance and insulin sensitivity by reducing adipocyte death in weight gain caused by high-fat diet intake in Male C57BL/6 mice (Defuria et al., 2009). Prior et al., indicated that ANTs fraction of blueberries significantly suppressed body fat accumulation and body weight gain in mice (Prior et al., 2010). Highbush Blueberry (*Vaccinium corombosum*) inhibited α-amylase and α-glucosidase activities *in vitro* and can be considered as an anti-diabetic drug (Johnson et al., 2011). In the study by Flores et al., acetonic extract of whole blueberry mitigated postprandial hyperglycemia via α-glucosidases inhibition (Flores et al., 2013).

ANT-enriched extracts of blueberries attenuated cardiac injury induced by cyclophosphamide *in vivo* condition via reduction of arterial blood pressure, and increases in enzyme activities and heart rate (Liu et al., 2015). It was shown that consumption of fresh blueberries for 75 days in a high cholesterol diet decreased the accumulation of cholesterol and oxidative stress in the guinea pig's aorta and liver. Consumption of blueberry was found to protect against oxidative stress and free radicals in red blood cells *in vivo* (Coban et al., 2013).

#### Strawberry *(Fragaria* × *ananassa)* Anthocyanins

Strawberry is a member of the Rosaceae family with abundant amounts of phenolic compounds, particularly ANTs and ellagic acid (Andersen et al., 2004). Strawberries contain different types of ANTs, such as ascyanidin 3-glucoside, pelargonidin 3-glucoside, pelargonidin3-rutinoside, pelargonidin 3-acetylglucoside, and cyanidin 3-rutinoside. Furthermore, 5 carboxypyranopelargonidin 3-glucoside and four purple ANT flavanol complexes consisting of pelargonidin 3-glucoside were detected in strawberries.

Some of the known cardioprotective agents in strawberries including vitamin C, folic acid, potassium, fiber and phytosterolscontribute to the antioxidant, anti-inflammatory, and hypocholesterolemic effects of these fruits (Wang and Lin, 2000).

ANTs in strawberries also reduced obesity in mice, inhibited esophageal cancer, suppressed ox-LDL-induced proliferation, reversed behavioral aging in rats and possessed anticarcinogenic and antithrombotic effects (Wang and Lin, 2000; Qin et al., 2009).

Ox-LDL, a marker of oxidative stress, is elevated in subjects with established coronary heart disease (CHD) and is a prognostic marker for the progression of subclinical atherosclerosis (Toshima et al., 2000).

The anti-hyperglycemic effects of Brazilian strawberries have been reported in *in vitro* model (da Silva Pinto et al., 2008) In mice models, freeze-dried strawberry powder was shown to reduce obesity and improved glycemic control in those fed a high-fat diet while ANT-fed mice demonstrated an upregulation of anti-inflammatory adiponectin gene. Serum cholesterol level was lowered following 4 weeks consumption of freeze-dried strawberries due to the presence of phytosterol, fiber, or other phytochemicals. Suppression of LDL-cholesterol as well as lipid peroxidation was also noted. The antioxidant rich phytochemicals in strawberries have been shown to reduce the central nervous system deficits caused by aging in rat models (Andersen et al., 2004). The cardiovascular health benefits of strawberries were also associated to the reduction of thiobarbituric acid-reactive substances in LDL and decrease in lipids oxidative damage in hyperlipidemic subjects. In addition, strawberries ANTs, such as pelargonidin-3-O-glucoside, reduced postprandial inflammation and increased insulin sensitivity in overweight adults (Wang and Lin, 2000).

#### Maqui Berry *(Aristotelia chilensis)* Anthocyanins

The fruit from *Aristotelia chilensis* (Molina) Stuntz, commonly known as Maqui Berry, Chilean blackberry or “maqui” in Chile and Argentina, is a common wildberry that belongs to the Elaeocarpaceae (Toshima et al., 2000). Maqui berry has recently been reported as one of the healthiest exotic berries due to its particularly high concentration of bioactive polyphenols (Schreckinger et al., 2010). Studies on the phytochemical composition of Maqui berry have confirmed the presence of phenolic acids, proanthocyanidins, and ANTs such as delphinidin-3- sambubioside-5-glucoside. The leaves and fruits of Maqui berry have been used in folk medicine to treat a variety of ailments including sore throat, kidney pains, ulcers, fever, inflammation, and diarrhea.

*In vitro* studies have demonstrated that Maqui berry significantly inhibits nitrite oxide production, which is comparable to the effect exerted by quercetin, a potent anti-inflammatory agent via inhibition of prostaglandin E2 and the COX-2 on LPS-stimulated RAW 264.7 macrophages (Morazzoni and Bombardelli, 1996; Schreckinger et al., 2010). In another *in vitro* study, the extract of Maqui berry suppressed the production of glucose and attenuated the downregulation of gluconeogenic enzyme and glucose-6-phosphatase. Moreover, oral administration of delphinidin 3-sambubioside-5-glucoside decreased the fasting blood glucose in obese C57BL/6J mice (Pergola et al., 2006; Rojo et al., 2012) and can be a therapeutic agent for managing MetS and diabetes.

The Maqui berry also showed cardioprotective effect against ischaemia–reperfusion heart damage in mice. Maqui berry possessed antioxidant activity and the highest oxygen radical absorbance capacity (ORAC) by inhibiting LDL oxidation and adipogenesis, also played a protecting role against intracellular oxidative stress in human endothelial cells (Schreckinger et al., 2010).

#### Black Chokeberry Anthocyanins *(Aronia melanocarpa)*

*Aronia melanocarpa* is one of the richest plant sources of polyphenolic substances, especially ANT glycosides with the highest antioxidant capacity among the polyphenol-rich beverages (Kulling and Rawel, 2008). Black chokeberry decreases weight gain, attenuates insulin resistance, reduces adipogenesis, and plasma concentrations of total cholesterol, LDL-cholesterol, and TGs. *In vitro* experiments showed that the phenolic constituents of black chokeberry exhibited anti-platelet effects as well as vasoactive and vasoprotective properties in porcine coronary arteries (D'alessandro et al., 2012). *In vivo* studies have shown that black chokeberry extract significantly exhibited hypoglycemic and antidiabetic effect by inducing the glucose uptake and glycogen synthesis, increasing insulin secretion, and protecting pancreatic β cells in streptozotocin (STZ)-induced oxidative stress in male wistar rats (Renaud and De Lorgeril, 1992; Valcheva-Kuzmanova and Belcheva, 2006; Jurgonski et al., 2008).

Qin and Anderson reported that diet supplemented with chokeberry extract reduced body weight gain significantly after 4 weeks via lipase and pancreatic amylase inhibition along with reducing carbohydrates absorption in the digestive system (Qin and Anderson, 2012).

Frejnage and Zduńczyk suggested that diets supplemented with 0.4, 0.8, and 1.2% of chokeberry extract suppressed the prooxidative activity *in vivo* by reducing blood malonylodialdehyde content in rats (Frejnagel and Zdunczyk, 2008). Olas et al. reported that ANTs of *Aronia* attenuated lipid peroxidation and possessed antioxidative activity in peroxynitrite induced stress *in vitro*. It may be helpful in managing the reduction-oxidation (redox) homeostasis disturbance by inhibiting nuclear factor (NF)-κB and increasing glutathione peroxidase activity, which confirms the beneficial effect of *Aronia melanocarpa* in patients with MetS and diabetes (Olas et al., 2008). Kim et al. reported that *Aronia* modulated hepatic lipid metabolism and improved antioxidant function in mice (Simeonov et al., 2002; Kim et al., 2013).

It has been found that black chokeberry extract significantly exhibited hypoglycemic and antidiabetic effect *in vivo* by induction of glucose uptake and glycogen synthesis and by elevating insulin secretion. It also helped to maintain the round shape of the pancreas and protected the pancreatic β cells in STZ-induced oxidative stress along with defeating sucrase and maltase activities in male wistar rats (Jeon et al., 2018).

#### Mulberry Anthocyanins

Mulberry contains water soluble ANTs, such as cyanidin-3-glucoside (47%), cyanidin-3-rutinoside (27%), and pelargonidin-3-glucoside (1.4%), which has been traditionally used in Chinese medicines. Mulberry was shown to have great antioxidant, anti-inflammatory, and anti-cancer activities in both cultured cells and animal models (Hassimotto et al., 2008; Huang et al., 2013). The dietary supplements with mulberry ANTs mitigate adverse effects of high-glucose against diet-induced obesity in C57BL/6 mice. Yan et al. demonstrated that mulberry ANTs reduced fasting blood glucose, serum insulin and leptin, as well-modulated TGs, cholesterol, and LDL values in high-fat diet in db/db mice (Yan et al., 2016).

#### Purple Sweet Potato *(Ipomoea batatas)* Anthocyanin

The sweet potato (*Ipomoea batatas*), is a dicotyledonous plant that belongs to Convolvulaceae family; it is large, starchy, and sweet-tasting, making it consumed as a food additive for the prevention and care of type 2 diabetes, anemia, and hypertension. Sweet potato contains a variety of ANTs. An *in vivo* study of sweet potato demonstrated that oral administration improved diabetes, glucose tolerance, hyperinsulinemia, and hyperlipidemia, also lowered TGs and free fatty acid in zucker fatty rats (Kusano and Abe, 2000). It also exhibited hypoglycemic activity in STZ- induced insulin deficiency in yellow db/db mice. Ludvik et al., observed a reduction in oral glucose insulin sensitivity following Caiapo treatment (Ludvik et al., 2008). Matsui et al. showed that *in vivo* oral administration of the diacylated ANTs derived from *I. batatas* in rats exhibited a potent maltase inhibitory activity, and significantly decreased maximal blood glucose level and serum insulin secretion compared to vehicle treatment (Matsui et al., 2002). It has also been shown that oral administration of Caiapo 4 g/day for 6 weeks lowered total and LDL cholesterol levels as well as blood glucose by increasing insulin sensitivity without affecting insulin secretion. Administration of *I. batatas* could significantly increase the level of adiponectin, which is produced by adipocytes and acts as a modulator of insulin sensitivity (Ludvik et al., 2008).

#### Pomegranate Seed Anthocyanins

Pomegranate (*Punica granatum*, Punicaceae) is an edible fruit comprising of 80% juice and 20% seed, and cultivated in Mediterranean countries, China, Japan, Russia and the United States. ANTs detected in pomegranate include pelargonidin 3-glucoside, cyanidin 3-glucoside, delphinidin 3-glucoside, pelargonidin 3,5-diglucoside, cyanidin 3,5-diglucoside, and delphinidin 3,5-diglucoside. The proanthocyanidins and ANTs of this plant were found to show antiangiogenic, antioxidant, anti-carcinogenic, and antimicrobial activities, besides, these compounds were shown to inhibit the activities of cyclooxygenase (COX), nitric oxide, and epidermal growth factor receptor (Bagchi et al., 2004; Vasconcelos et al., 2006).

Studies have reported that pomegranate fruit extract demonstrated anti-inflammatory activitiy by modulating the production of prostaglandin and leukotriene along with inhibition of COX and lipoxygenase. α-Tocopherol from seeds of this plant inhibited sphingolipid synthesis and COX-2 activity. Recent studies have shown that pomegranate wine can inhibit NF-κB in vascular endothelial cells. Dietary utilization of pomegranate juice significantly diminished the atherosclerotic lesions formation and decreased LDL oxidation in atherosclerotic mice (Schubert et al., 1999; Aviram et al., 2000; Gil et al., 2000; Aviram and Dornfeld, 2001; Kaplan et al., 2001; Chidambara Murthy et al., 2002). It has shown that pomegranate seed oil can reduce weight gains and food consumption in male rats fed a high-cholesterol diet. Vroegrijk et al. also observed a significant reduction in body fat mass in male C57BI/J6 mice fed with a high-fat diet (Vroegrijk et al., 2011).

#### Red Onions *(Allium cepa)* Anthocyanins

Red onions (*Allium cepa*), a widely consumed vegetable with purplish-red skin which comes from anthocyanidins such as cyanidin, belongs to Liliaceae family native of Southwest Asia, and is widely cultivated throughout the world. Red onions are an abundant source of flavonols, including quercetin derivatives, such as quercetina glycine, its glycosides, and ANTs (Kaplan et al., 2001). Onion significantly decreased the levels of total cholesterol and LDL, and attenuated hypertension and blood cholesterol in diabetic animal models (Kumari and Augusti, 2002). Red onion supplementation attenuated high-fat diet-induced insulin resistance in C57BL/6J mice by limiting adiposity and increasing energy expenditure (Eldin et al., 2010; Jung et al., 2011).

Several *in vivo* studies showed that onion ingestion improves hyperglycemia in diabetic patients via increasing insulin sensitivity, improving glucose tolerance, and protecting DNA from oxidative stress in mice (Mathew and Augusti, 1975; Corzo-Martínez et al., 2007). Morrison et al. explained that the reduction of obesity and improvement of insulin sensitivity might be related to the upregulation of energy expenditure and biogenesis of mitochondrial skeletal muscle in C57BL/6J mice upon red onion extract supplementation (Morrison et al., 2015). These reports confirmed the therapeutic effects of *A. cepa* in patients with MetS and diabetes.

### Purified Anthocyanins

#### Cyanidin 3-Glucoside (C3G)

The most common anthocyanidin, cyanidin, is present in 90% of fruits. It is absorbed into blood circulation in an intact form and metabolized to methoxy derivatives in the liver and kidney, and its metabolites may modulate metabolic effects. Studies have shown that the antioxidant activity of cyanidin was more than that of vitamin E and Trolox, and was comparable to that of synthetic antioxidants, such as tert-butylhydroquinone (TBHQ), butylated hydroxytoluene (BHT), and butylated hydroxyanisole (BHA) likely because of free hydroxyl groups on the 3′ and 4′ positions of cyanidin (Amorini et al., 2001).

It has been proven that C3G has antioxidative and anti-inflammatory properties based on *in vitro* and *in vivo* studies. C3G significantly suppressed the development of high-fat diet induced obesity C57BL/6 mice and modulated the gene expression of adipocytokines in human adipocytes, and reduced inflammation and adipocyte death, but not adipocyte size in high-fat diet mice *in vivo* (Tsuda et al., 2003). C3G also diminished inflammation in isolated vascular endothelial cells and monocytes *in vitro* and possessed an insulin-like effect in human omental adipocytes and 3T3-L1 cells. Attenuation of gene expression of adipocytokines is also seen in human adipocytes. Other studies have reported that C3G or its aglycone induced upregulation of adiponectin, which enhanced insulin sensitivity in isolated rat and human adipocytes, but these events were not observed *in vivo*. C3G efficiently inhibited free fatty acids (FFAs) and glycerol release from the adipocytes during hyperglycemia in high glucose-induced lipolysis in cultured 3T3-L1 adipocytes. It also increased the activity of AMPK and decreased the activity of glutamine, fructose 6-phosphate, and aminotransferase. C3G reduced hyperglycemia-promoted *O*-glycosylation of transcription factor Foxo1, resulting in decreased expression of adipose triglyceride lipase. Purple corn is a source of C3G, and has been shown to decrease body fat and hyperglycemia in high-fat diet mice. In another study, C3G increased adipocyte glucose uptake and GLUT4 membrane translocation significantly. Nuclear PPARγ activity was increased as well as adiponectin (Scazzocchio et al., 2011). C3G isolated from mulberry fruits possesses an antidiabetic effect via decreasing oxidative stress, and increasing antioxidant defense system and cytoprotective activity during glucose-induced apoptosis in MIN6N pancreatic β-cells by depleting generation of ROS, DNA fragmentation, and the rate of apoptosis (Lee et al., 2015). Pure C3G also increases in cholesterol efflux, ABCA1 expression, PPARa, LXRa, and decreases in proinflammatory molecules, such as ICAM1, MCP1, TGFb1, and NF-κB in HK-2 cells (Du et al., 2015). Zhang et al. suggested that Ik-Ba and NF-κB nuclear translocation have a significant role in therapeutic effects of C3G (Zhang et al., 2010). C3G significantly suppressed body fat accumulation induced by a high-fat diet, which is attributed to a reduction in lipid synthesis in the liver and white adipose tissue in C57BL/6J mice. It has also been demonstrated that C3G significantly ameliorated hyperglycemia and insulin sensitivity *in vivo* (Tsuda et al., 2003). A recent *in vivo* study reported that C3G inhibited release of purified platelet granule and protected against CVD and thrombosis (Zhou et al., 2017). Furthermore, it also diminishes blood glucose level and modulates insulin sensitivity in type 2 diabetic mice. It has also been proven that C3G had synergistic effect with acarbose, a inhibitor of α-glucosidase used in the treatment of diabetes. Furthermore, C3G treatment resulted in increased insulin secretion compared with the control diabetic group, and is a potential phytotherapeutic agent for the prevention of diabetes (Zhou et al., 2017).

Cyanidin-3-glucoside and peonidin-3-glucoside in black rice may decrease antioxidant and anti-inflammation activity by protecting against oxidative damage and suppressing nitric oxide synthase in mouse macrophage cell linings (Hu et al., 2003).

#### Pelargonidin

Lamy et al., showed that delphinidin inhibited phosphorylation of vascular endothelial growth factor (VEGF) receptor-2 in human umbilical vascular endothelial cells (Tonelli et al., 2009). *In vitro* studies showed that a pelargonidin derivative enhanced insulin secretion by β-cells and could be a good anti-diabetic agent via suppression of fasting blood glucose level almost to half of the pretreatment levels. Furthermore, urine sugar decreased to (non-significant/minor) traces and appeared healthy (Cherian et al., 1992) NF-κB.

*In vivo* studies showed that pelargonidin significantly ameliorated the alteration in hyperalgesia through attenuation of oxidative stress in STZ -diabetic neuropathic rat. This compound also diminished diabetes-induced thiobarbituric acid reactive substances formation and reduce antioxidant defensive enzyme superoxide dismutase (Mirshekar et al., 2010). Roy et al., reported that pelargonidin normalized elevated blood glucose levels, improved serum insulin levels, decreased catalase and SOD, and enhanced fructosamine and malondialdehyde levels in diabetic rats (Roy et al., 2008).

#### Peonidin and Malvidin

Consumption of berries containing (57% malvidin and 33% petunidin or peonidin) was effective to reduce high-fat diet induced metabolic damage through individual significant effects on energy expenditure and increased activity by decreasing mitochondrial respiration and dissipation of the mitochondrial proton gradient (proton leak) in adipose tissue in C57BL/6 mouse model of polygenic obesity (Skates et al., 2017). Bognar et al. demonstrated that malvidin attenuated LPS-induced NF-κB, activation of mitogen activated protein kinase (MAPK), poly ADP-ribose polymerase, production of ROS, and depolarization of mitochondria (Bognar et al., 2013).

## Clinical Studies Confirming the Beneficial Effects of ANTs in MetS

ANTs are generally considered as safe remedy without considerable adverse effects and a wide range of pharmacological activities. Studies showed that ANTs have explicit useful effects on MetS features. However, studies on the effects of ANTs in prevention and treatment of MetS in human subjects are limited. The following studies demonstrated that regular consumption of ANTs diet may show protective effects in management and prevention of MetS.

In a systematic review and meta-analysis of 32 clinical studies, it was revealed that ANT-rich food can exert promising preventive and protective effects against cardiometabolic disorders. ANTs substantially decreased glycemic control markers, enhanced fasting and 2-h postprandial glucose level, and possessed favorable effects on controlling the LDL level (Yang et al., 2017).

Two-month *Aronia* extract therapy resulted in considerable decline in systolic and diastolic blood pressure, besides suppressed the LDL, TGs, and Endothelin-1 in 47 subjects (32 women, 15 men). Moreover, *Aronia* fruit juice reduced the elevated cholesterol, LDL, and plasma lipids concentration (Broncel et al., 2007).

A randomized controlled study on 48 participants with MetS, who have been treated with freeze-dried and fresh blueberries for 8 weeks daily exposed that blueberry beverage significantly decreased the plasma oxidized LDL, diastolic, and systolic blood pressure, also ameliorated the serum malondialdehyde and hydroxynonenal levels (Basu et al., 2010).

Regular chokeberry juice drinking (250 mL per day) was recorded to decrease LDL and TGs, increased HDL cholesterol level and led to significant reduction in glucose serum, homocysteine and fibrinogen in men with mild hypercholesterolaemia (Skoczynska et al., 2007). Black chokeberry also decreased glucose concentration and fasting blood glucose in human studies. In addition, it may also diminish oxidative and/or nitrative stress that occurred in platelets from breast cancer patients (Olas et al., 2008). Maqui Berry ANTs consumption in subjects with hyperlipidemia and dyslipidemia depressed LDL and VLDL and increased HDL cholesterol (Alvarado et al., 2016). Delphinol is a proprietary Maqui berry extract with a standardized content of 25% w/w delphinidin glycosides and 35% total ANTs that can significantly inhibit postprandial blood glucose (Hidalgo et al., 2014).

In a factorial randomized design study, 1-month therapy with *Hibiscus sabdariffa* extract powder significantly enhanced HDL-c levels, amended the ratio of TAG/HDL-c, reduced glucose and total cholesterol levels as well as triglycerides in MetS patients (Gurrola-Díaz et al., 2010).

In another randomized controlled trial on 27 subjects with MetS, 4 cups of freeze-dried strawberry beverage daily for 8 weeks, caused hypocholesterolemic effects in study subjects through decreasing the total and LDL-cholesterol levels along with suppression of VCAM-1 circulating levels (Basu et al., 2010). Another study on eight elderly women exhibited that consumption of strawberries, red wine, spinach, or vitamin C can increase human serum antioxidant capacity (Cao et al., 1998).

The simultaneous consumption of blackcurrant ANTs and apple polyphenols was the subject of a clinical study on five postmenopausal women and 20 men, investigating the effect of this mixture completed with a meal containing starch and sucrose on the initial postprandial glycemic response. The mixture was found to be effective in inhibition of the early responses (0–30 min) of plasma glucose and insulin, and reduction of postprandial glycemia. Insulin and incretin excretion were reduced as the secondary results. The promising inhibitory role of ANT and proanthocyanidin-riched diets on the negative effects of high-carbohydrate meals was highlighted by this study (Castro-Acosta et al., 2017). Modulation of lipid and glucose-metabolism, antioxidative, and anti-inflammatory activities were the other outcomes of the inclusion of ANTs in human diets. These findings have been corroborated in an investigation carried out by Kim et al., who chosed Açaí berries as a rich source of ANTs to be consumed by 37 subjects (12 weeks) suffering from MetS (Kim et al., 2018). In result, the plasma levels of interferon gamma (IFN-γ) and urinary level of 8-isoprostane were decreased. However, all parameters related to the glucose and lipid metabolisms were found to be unchanged after intake of the beverage (Kim et al., 2018). Although their study verified the health-promoting effect of ANT-rich diets in metabolically challenged humans, further clinical investigations are required to warranty these specific results.

A search in *www.clinicaltrials.gov* has shown that many completed and ongoing clinical trials are evaluating the therapeutic potential of ANTs for the treatment of fatty liver disease, CVD, MetS, coronary artery disease, and type 2 diabetes. Retrieved clinical trials registered in *www.clinicaltrials.gov* are summarized in Table 4.

**Table 4:** Completed and ongoing clinical trials.

**Number**	**Title**
NCT02407522	The improvements of dietary supplement of black rice on MetS (IDSBRMS)
NCT02999256	Effect of cherry juice on fat oxidation and cardio-metabolic markers
NCT01399138	The effect of blueberry powder supplementation on cardiovascular risk factors in subjects with the MetS
NCT00992641	The effect of nordic recommended diet on the features of MetS
NCT01562392	Effects of berries and vegetables on cardiometabolic risk markers and cognitive function
NCT01414647	The health effect of diet rich in nordic berries (berry)
NCT01224743	Effect of fruit and vegetable concentrates on endothelial function in persons with MetS
NCT01154478	Effects of dietary polyphenols and ω-3 fatty acids on cardiovascular risk factors in high risk subjects (Etherpaths)
NCT02035592	The health effects of blueberry ANTs in MetS (ongoing)
NCT01766570	Beneficial effects of a polyphenol enriched beverage on type 2 diabetes prevention and on cardiovascular risk profile of men and women with insulin resistance.
NCT01245270	A single supplement of a standardized bilberry extract modifies glycaemic response
NCT01180712	Study of oral ants on insulin resistance
NCT01860547	Effects of berries and berry fractions on metabolic diseases
NCT02689765	Effect of ants on metabolic profiles in subjects with pre-diabetes
NCT02779985	Goji berries and energy expenditure
NCT02017132	Effect of pomegranate extract intake on body composition and blood pressure.
NCT01568983	The effects of polyphenol-rich berry juice on blood pressure in hypertensive subjects
NCT02459756	Ant-rich blackcurrant and vascular function

## Conclusion and Future Prospects

MetS is closely related to obesity and has a major role in initiating CVD and many other pathological complications of type 2 diabetes.

**Figure 3:**
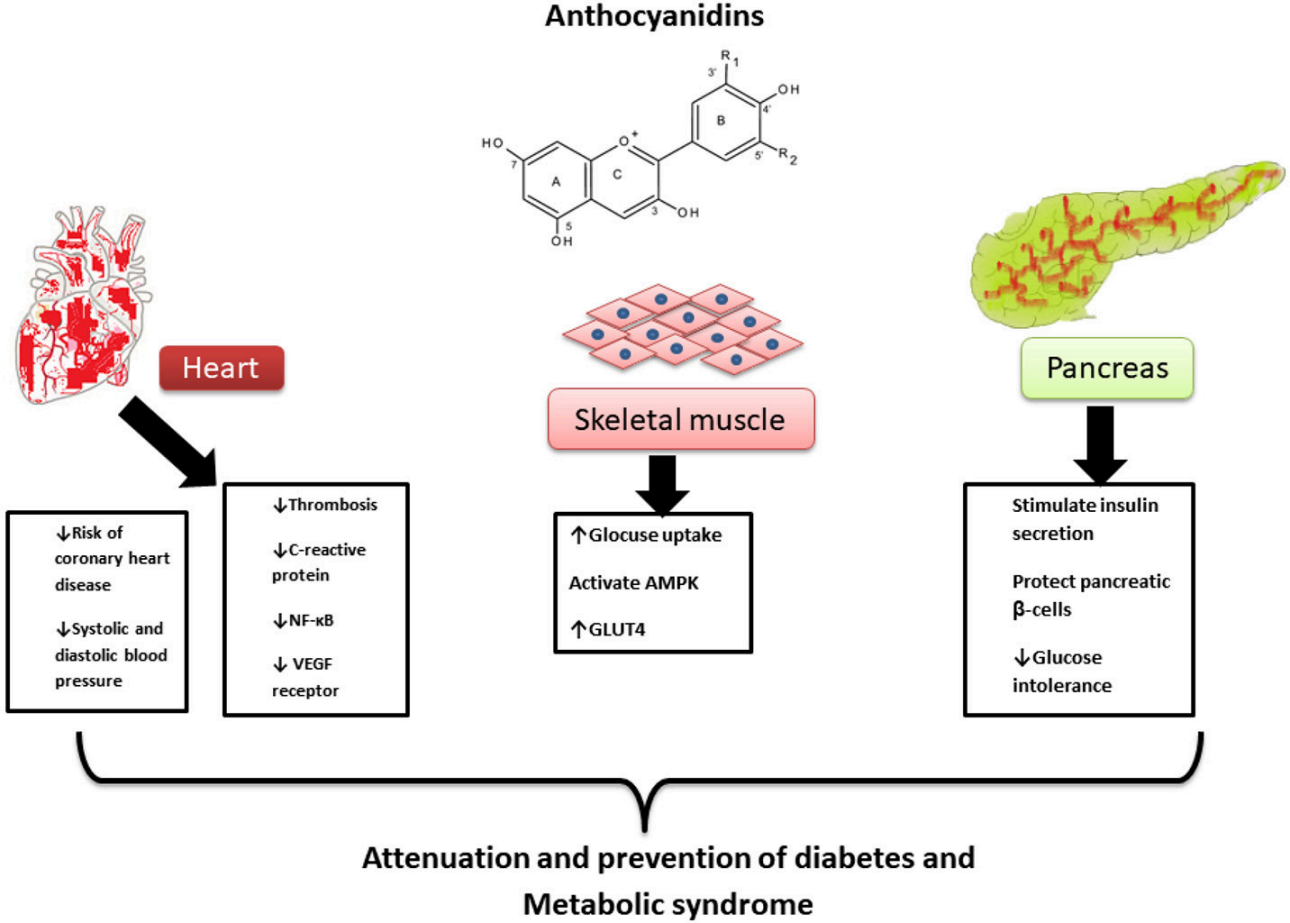
Pharmacological mechanisms of anthocyanidins in metabolic syndrome.

Diet has an important role in disease management and prevention. ANTs have antioxidant properties and possess a protective role for pancreatic β-cells from glucose-induced oxidative stress. They also act as anti-inflammatory and hypotensive agents (Lietti et al., 1976). Additionally, it has been reported that ANTs cause a reduction in concentrations of TC, LDL-C, and TGs, as well suppress the expression of enzymes responsible for fatty acid synthesis. ANTs protect against CVD, cancer and diabetes; also attenuate the symptoms of MetS such as dyslipidaemia, insulin resistance, impaired glucose tolerance, hypertension, hyperglycemia, and glucosuria. ATNs also inhibit the activity of α-glucosidase against maltase and sucrose, and increase the excretion of insulin in primary cell culture. ANT-rich extracts showed a lowering effect on plasma lipid profiles in rodent models of hyperlipidaemia. ANTs in chokeberry and purple maize reduced visceral adiposity, systolic blood pressure, and total body fat, moreover, reduced the glucose tolerance, liver, and cardiovascular structure and function. Figure 3 shows the main pharmacological mechanisms of ANTs in MetS.

This review focused on a group of dietary ANTs, and their positive effect in human health. Our presentation demonstrated that more investigations into the efficacy and intracellular mechanisms of dietary ANTs are necessary to recognize the metabolism, bioefficacy, and main mechanisms of action in MetS. Current evidence establishes that dietary ANTs and its pharmaceutical supplements can be considered as a functional nutritional supplement for the prevention and management of metabolic syndrome and its complications.

## Author Contributions

RN and FF did a literature review and prepared the first draft of the manuscript. PH, SN, SH, DT, and SM edited the manuscript and proposed/included some vital modifications. MF and RK design throughout the work and did the final edition of the manuscript.

### Conflict of Interest Statement

The authors declare that the research was conducted in the absence of any commercial or financial relationships that could be construed as a potential conflict of interest.
